# The influence of sexual activity on the vaginal microbiota and *Gardnerella vaginalis* clade diversity in young women

**DOI:** 10.1371/journal.pone.0171856

**Published:** 2017-02-24

**Authors:** Lenka A. Vodstrcil, Jimmy Twin, Suzanne M. Garland, Christopher K. Fairley, Jane S. Hocking, Matthew G. Law, Erica L. Plummer, Katherine A. Fethers, Eric P. F. Chow, Sepehr N. Tabrizi, Catriona S. Bradshaw

**Affiliations:** 1 Melbourne Sexual Health Centre, Alfred Health, Carlton, Melbourne, Australia; 2 Central Clinical School, Faculty of Medicine, Nursing and Health Sciences, Monash University, Melbourne, Australia; 3 Centre for Epidemiology and Biostatistics, Melbourne School of Population and Global Health, The University of Melbourne, Parkville, Victoria; 4 Department of Molecular Microbiology, Murdoch Children’s Research Institute, Melbourne, Australia; 5 Department of Microbiology and Infectious Diseases, The Royal Women’s Hospital, Melbourne, Australia; 6 Department of Obstetrics and Gynaecology, University of Melbourne, Melbourne, Australia; 7 The Kirby Institute, University of New South Wales, Darlinghurst, Australia; 8 Department of Microbiology, The Royal Children’s Hospital, Melbourne, Australia; Fred Hutchinson Cancer Research Center, UNITED STATES

## Abstract

**Objectives:**

To examine the influence of sexual activity on the composition and consistency of the vaginal microbiota over time, and distribution of *Gardnerella vaginalis* clades in young women.

**Methods:**

Fifty-two participants from a university cohort were selected. Vaginal swabs were self-collected every 3-months for up to 12 months with 184 specimens analysed. The vaginal microbiota was characterised using Roche 454 V3/4 region 16S rRNA sequencing, and *G*.*vaginalis* clade typing by qPCR.

**Results:**

A *Lactobacillus crispatus* dominated vaginal microbiota was associated with Caucasian ethnicity (adjusted relative risk ratio[ARRR] = 7.28, 95%CI:1.37,38.57,*p* = 0.020). An *L*.*iners* (ARRR = 17.51, 95%CI:2.18,140.33,*p* = 0.007) or *G*.*vaginalis* (ARRR = 14.03, 95%CI:1.22,160.69, *p* = 0.034) dominated microbiota was associated with engaging in penile-vaginal sex. Microbiota dominated by *L*.*crispatus*, *L*.*iners* or other lactobacilli exhibited greater longitudinal consistency of the bacterial communities present compared to ones dominated by heterogeneous non-lactobacilli (*p*<0.030); sexual activity did not influence consistency. Women who developed BV were more likely to have clade GV4 compared to those reporting no sex/practiced non-coital activities (OR = 11.82, 95%CI:1.87,74.82,*p* = 0.009). Specimens were more likely to contain multiple *G*.*vaginalis* clades rather than a single clade if women engaged in penile-vaginal sex (RRR = 9.55, 95%CI:1.33,68.38,*p* = 0.025) or were diagnosed with BV (RRR = 31.5, 95%CI:1.69,586.87,*p* = 0.021).

**Conclusions:**

Sexual activity and ethnicity influenced the composition of the vaginal microbiota of these young, relatively sexually inexperienced women. Women had consistent vaginal microbiota over time if lactobacilli were the dominant spp. present. Penile-vaginal sex did not alter the consistency of microbial communities but increased *G*.*vaginalis* clade diversity in young women with and without BV, suggesting sexual transmission of commensal and potentially pathogenic clades.

## Introduction

There is limited information on how commencement of sexual activity affects the composition and stability of the vaginal microbiota. In vaginal samples from women collected pre and post self-reported penile-vaginal sexual debut, initiation of penile-vaginal sex increased detection of *Gardnerella vaginalis* by quantitative polymerase chain reaction (PCR) [1]. However, colonization by other bacterial species was not altered. Non-coital sexual activities were not separately investigated. The vaginal microbiota composition in peri-menarchal adolescents is reportedly similar to those of sexually active reproductive-age women [2].

The bacterial classification of the vaginal microbiota of reproductive-age women has been described for different populations and is dependent on various factors including population size and technology used [3]. Initially Ravel and colleagues described five community state types (CSTs) dominated by either *Lactobacillus crispatus* (CST I), *L*. *gasseri* (CST II), *L*. *iners* (CST III), *L*. *jensenii* (CST V), or heterogeneous non-lactobacilli (CST IV; further delineated into two sub-groups IVA and IVB) [4, 5]. In the largest study of the vaginal microbiota to date, Fettweiss *et al* [6] identified the above five groups among women without BV. In addition, they identified two groups dominated by *G*. *vaginalis* or BV associated bacteria type I (BVAB1), with differences in the spp. colonizing the vaginal microbiota being reported for various ethnic groups. Although the actual number of vaginal community types is still under discussion, differences in ethnicity may explain why some women have a vaginal microbiota more prone to change over a short time. Although lactic acid producing bacteria including *L*. *crispatus* and *L*. *gasseri* have been associated with the most stable vaginal microbiota [5].

In vaginal communities lacking lactobacilli, anaerobic bacteria predominate. In Ravel’s study, *G*. *vaginalis* was the most common spp. identified in CST IV [7], a CST commonly associated with the clinical syndrome bacterial vaginosis (BV). Although *G*. *vaginalis* appears to be universally present (in high copy numbers) in women with BV, it is also detected, albeit at lower yields, in asymptomatic women [7]. Comparative genome analysis of *G*. *vaginalis* isolates has revealed at least four genetically distinct clades [8], with one study suggesting *G*. *vaginalis* clade (GV)1 and GV3 are more common in BV [9]. *G*. *vaginalis* clade type in young women, particularly with differing sexual experience, has not been described.

We aimed to determine how sexual activity affects the composition and stability of vaginal bacterial communities and *G*. *vaginalis* clade types present in young women who participated in a female university student study (FUSS), in Melbourne, Australia [10, 11].

## Methods

Participants and specimens for this study were selected from the original FUSS cohort comprising 413 female university students aged 17–21 without BV at enrolment [10]. Each participant completed demographic and behavioural questionnaires (including sexual and contraceptive behaviours), self-collected vaginal swabs and smears every three months for up to 12 months. Smears underwent blinded Nugent scoring (NS; graded as: normal flora NS = 0–3, intermediate flora NS = 4–6, or BV NS = 7–10) [12] by two microscopists and swabs were rotated in 1ml RNAlater (Life Technologies; Thermo Fisher Scientific, Waltham, USA) and stored at -80C until analysis. The study endpoint was incident BV (NS = 7–10) or reaching 12 months without BV.

Participants were eligible for inclusion if they provided complete behavioural and microbiological data every three months (follow-up interval) from recruitment until endpoint. Specimens were selected based on which past and ongoing sexual activity category the participants belonged to over the 12 month study period. At least one act of penile-vaginal sex had to be unprotected (no condoms used) to be selected for the applicable category. The few women in our study who reported complete intervals of 100% protected penile-vaginal sex were included as having practiced non-coital sex (all reported either digital and/or oral sex).

**Fig 1:**
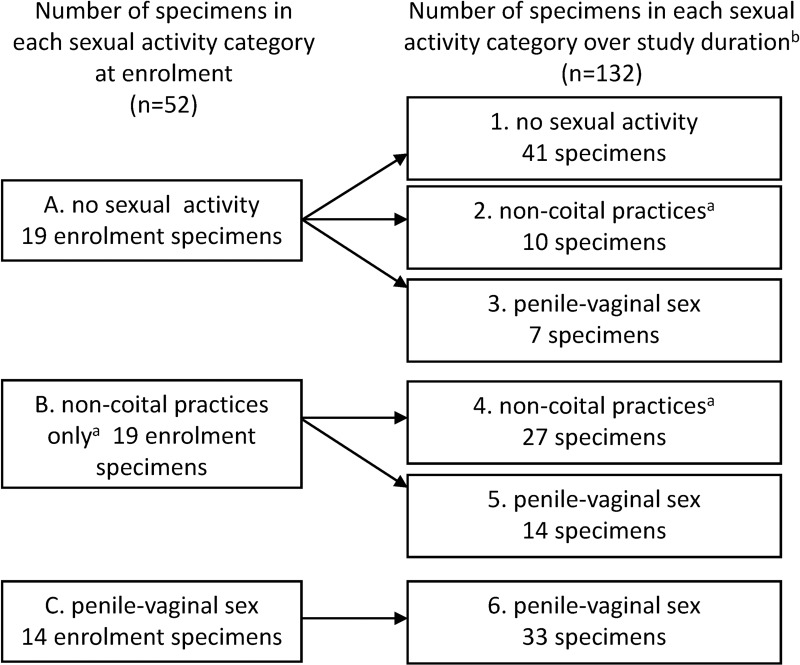
Flow chart of number of specimens assessed according to sexual activity categories at enrolment and over the study duration. **Total specimens = 184. At least one act of penile-vaginal sex had to be unprotected (no condoms used) to be selected for the applicable categories.** ^a^includes women engaging in digital or receptive oral sex or 100% condom use for penile-vaginal sex in addition to these practices; ^b^the number of specimens shown for each category of sexual activity are from women who remained static within a category or progressed to a new category.

Initially there were three sexual activity categories at enrolment: A) those with no past sexual activity, B) those that had only ever practiced non-coital sex, and C) those who had engaged in at least one episode of unprotected penile-vaginal sex (Fig 1). From these women, specimens were selected to represent the following six overall sexual activity categories: 1) women with no past sexual contact with others and no sexual contact over 12 months, 2) women with no past sexual contact with others who commenced non-coital sex, and/or 3) at least one episode of unprotected penile-vaginal sex for the first time during the 12 months, 4) women with a past history of non-coital practices who continued non-coital practices over 12 months, or 5) transitioned from non-coital sex to unprotected penile-vaginal sex over the study, and 6) women with a past history of unprotected penile-vaginal sex who continued to engage in penile-vaginal sex (Fig 1). We selected up to 10 eligible participants from each of these six categories but were limited by the number of participants fulfilling each category criteria. We selected all participants in categories containing <11 women and used a random number generator to select participants if there were ≥11 women in a category. The BV incidence rate in this cohort was strikingly low (2.2/100 woman-years[WY]; 95%CIs: 0.8, 4.9/100WY) [10, 11] and in addition to the specimens selected, we included all of the specimens from the seven woman from the cohort who had a BV diagnosis by Nugent score. Specimens from women who developed BV in the cohort (n = 7) were analysed within their relevant sexual behaviour groups, ethnic groups or according to OCP-use for all analyses, with the exception of the *G*. *vaginalis* clade analysis, where they were analysed as a separate group (see below). This resulted in the inclusion of 52 women and a total of 184 longitudinal specimens (Fig 1).

### Ethics, consent and permissions

The University of Melbourne Human Research and Ethics Committee approved this study (ID: 0719743) [10]. Women provided written consent to participate and gave permission for their specimens to be used for the current study.

### Experimental procedures

Genomic DNA was extracted as previously described [7]. Specimens were barcoded with a multiplex identification tag (10bp) during PCR using primers targeting the V3/4 16S rRNA gene region (341F/805R) [13] and underwent pyrosequencing using the Roche 454 Genome Sequencer FLX Titanium Chemistry (Macrogen Inc. Seoul, South Korea). Taxonomic assignment of each read was carried out using MG-RAST (Sequences are available from the MG-RAST server under project ID 1224 [http://metagenomics.anl.gov/linkin.cgi?project=mgp1224]) with an identity match of ≥98% and 200bp against the RDP database. In order to examine potential primer bias, the first 104/184 (56.5%) specimens analysed from 29 participants were screened using the primer set 27F and 338R targeting the V1/2 16S rRNA gene regions [14].

The bacterial abundances measured by V3/4 directed sequencing for *L*. *crispatus*, *L*. *iners* and *G*. *vaginalis* were compared to quantitative (q)PCR data for validation. qPCR assays for *L*. *crispatus* and *G*. *vaginalis* were used as described previously [7], and *L*. *iners* using the oligonucleotides and conditions described by Srinivasan *et al*. [15]. Presence of each of the four *G*. *vaginalis* clades were determined using multiplex qPCR as described by Balashov *et al*. [9], targeting unique sequences to each clade. Clade *G*. *vaginalis* (GV)1 was detected by unique features contained within the *G*. *vaginalis fuc1* gene (putative a-L-fucosidase; GI:311113989), and GV2, 3, and 4 by targeting a hypothetical protein of unknown function (GI:388060098), thioredoxin (GI:388062216), or a chloride transporter (GI:283783343), respectively.

#### Analyses

Demographic details were compared between sexual activity groups using the chi-squared test. Relative abundances and generation of Bray-Curtis dissimilarity scores were carried out using PRIMER-E V6 (PRIMER-E Ltd, Ivybridge, UK). A heat map using the Bray-Curtis distance measure was generated using R Studio (V0.98.1103, Boston, USA) employing R3.2.0. The heatmap.2 function in gplots was used to draw the heat map.

Statistical analyses were performed using Stata/IC (Version 14, StataCorp LP, College Station, USA). Univariate multinomial logistic regression examined the effect of ethnicity, OCP-use or sexual activity on the dominant bacterial spp. present in a specimen, clustering for multiple specimens per participant (52 participants/clusters). The relative risk ratio (RRR) represents the odds of having a vaginal microbiota being dominated by each of the specific *Lactobacillus* spp. or *G*. *vaginalis vs* one dominated by the referent group (other heterogeneous non-lactobacilli) simultaneously. Multivariate multinomial logistic regression examined the effect of all risk factors on the dominant bacterial spp. detected, presented as an adjusted (A)RRR, clustering for multiple specimens per participant.

Bray-Curtis dissimilarity scores were calculated between consecutive paired longitudinal specimens over multiple time points from each participant. Scores were given a value from 0–1; samples with a score of 0 being having all species/genera in common and those scoring 1 with no species/genera in common. Dissimilarity score statistics between pairs of specimens dominated by each bacterial group were summarised in boxplots. A random-effects linear model tested for differences in dissimilarity values between women with respect to ethnicity, OCP-use, sexual activity and dominant vaginal bacterial spp. present, clustering for multiple pairs per participant.

Logistic regression examined the distribution of clade types in specimens from women who had or had not engaged in penile-vaginal sex, clustering for multiple specimens per participant. Multinomial logistic regression examined the effect of sexual activity or development of BV on the number of different *G*. *vaginalis* clades present in a specimen, also clustering for multiple specimens (32 clusters). Specimens from women with no history of sexual activity or non-coital activities only were the reference group in this composite outcome comparing the RRR of number of *G*. *vaginalis* clades (3 *vs* 1 and 2 *vs* 1) simultaneously.

## Results

The mean age of the 52 women included in this study was 19.5 (±1.2SD), with 29 (56%) of Caucasian ethnicity, 19 (37%) Central and South-East Asian and 4 (8%) Indian/Sri-Lankan (Table 1). Smoking (4%) and douching (10%) were uncommon and 27% of participants reported currently using the oral contraceptive pill (OCP, Table 1). Caucasian women (*p* = 0.057) and OCP-users (*p*<0.001) were more likely to report that they had engaged in penile-vaginal sex in the last 12 months. Women with Central and South-East Asian ethnicity were more likely to report no prior sexual activity than to have engaged in either non-coital or coital sex (*p*<0.012). There was no association between other characteristics assessed.

**Table 1:** Demographic characteristics among 52 selected participants stratified according to their sexual activity category at enrolment.

	Sexual activity category at enrolment				
	no sexual activity	non-coital sexual activities only^a^	penile-vaginal sex	Total	
	N = 19	N = 19	N = 14	N = 52	
	n (%)	n (%)	n (%)	n (%)	*p* value
Age [mean(±SD)]	[19.2 (1.1)]	[19.4 (1.1)]	[20.2 (1.2)]	[19.5 (1.2)]	0.043^b^
Smoker	0	1 (5.3)	1 (7.1)	2 (3.9)	0.728^c^
Practices douching	3 (15.8)	2 (10.5)	0	5 (9.6)	0.417^c^
Current OCP use	4 (21.1)	0	10 (71.4)	14 (26.9)	**<0.001**^c^
***Ethnicity***					
Caucasian	7(36.8)	11 (57.9)	11 (78.6)	29 (55.8)	**0.057**^d^
Central & South-East Asian^e^	12 (63.2)	5 (26.3)	2 (14.3)	19 (36.5)	**0.012**^d^
Indian/Sri-Lankan	0	3 (15.8)	1 (7.1)	4 (7.7)	0.184^d^

All variables are self-reported. Key: SD = standard deviation, OCP = oral contraceptive pill.

^a^digital or receptive oral sex

^b^analysis of variance (ANOVA) of age by sexual activity category

^c^Fisher’s exact or Chi-squared analysis across all three sexual activity categories

^d^Chi-squared analysis comparing each ethnicity with the other two across all sexual activity categories

^e^comprises women of Chinese, Malay and Vietnamese ethnicities. The larger FUSS cohort consisted of N = 413 participants.

### Microbiota analyses

Fifty-two participants provided 184 longitudinal specimens for microbiota analysis, which were classified by self-reported sexual activity in the three months preceding each specimen collection (Fig 1). Nineteen women with no sexual activity at enrolment provided 77 specimens, 19 with non-coital activity only at enrolment provided 60 specimens, and 14 who reported penile-vaginal sex provided 47 specimens. This included the seven women who were diagnosed with BV on their endpoint specimen who provided a total of 22 specimens; 15 pre-BV specimens and 7 with BV diagnosed using the Nugent’s method.

#### Vaginal bacterial communities in the study population

All 184 specimens contributed to this analysis and a total of 827,833 high-quality 16S rRNA reads spanning the V3/4 region were assigned a taxonomic identity (mean = 4499 sequences per specimen; 95%CI: 4047,4951). A sub-analysis (*data not shown*) spanning the V1/2 region of the 16S rRNA gene within a subset of 25 participants (92 specimens) revealed no additional bacterial species; however *G*. *vaginalis* was under-represented in this subset, as described previously [14]. To validate the abundances measured by V3/4 directed sequencing, bacterial abundances for *L*. *crispatus*, *L*. *iners* and *G*. *vaginalis* were compared to qPCR data and demonstrated a strong correlation (*r* = 0.939, 0.931 and 0.845, respectively; *data not shown*).

**Fig 2:**
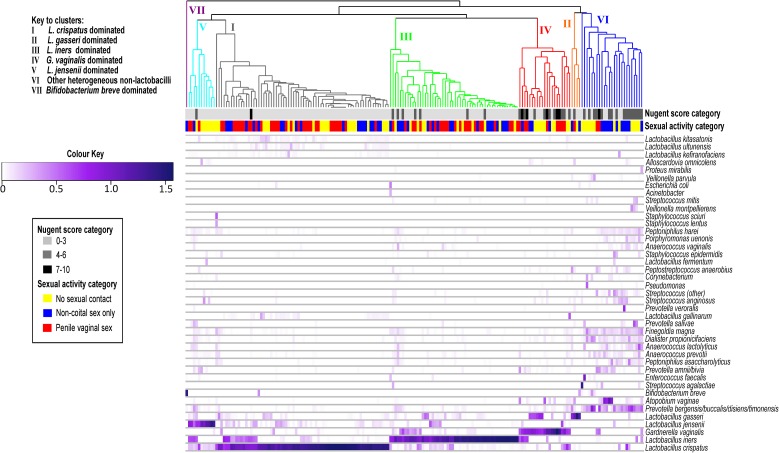
Heat map depicting vaginal bacterial communities analysed in this study. Heat map of proportions of bacterial taxa identified from 184 specimens from 52 participants. Values are expressed as arcsine transformed relative abundances. Nugent score categories and sexual activity categories for each specimen are indicated. Community state type (CST) groupings shown based on previous literature [4, 6, 16], where CST I aligns with specimens dominated by *Lactobacillus crispatus*, CST II with specimens dominated by *L*. *gasseri*, CST III with specimens dominated by *L*. *iners*, CST IV with specimens dominated by *G*. *vaginalis* and CST V with specimens dominated by *L*. *jensenii*. CST VI represents specimens dominated by other heterogeneous non-lactobacilli (predominantly *Atopobium vaginae* and *Prevotella* spp.) and CST VII specimens dominated by *Bifidobacterium breve*.

Specimens clustered into seven bacterial groups dominated by a specific *Lactobacillus* spp., *G*. *vaginalis*, *Bifidobacterium breve* or by other heterogeneous non-lactobacilli spp. (Fig 2). The bacterial communities amongst all 184 specimens were primarily dominated by *L*. *crispatus* or *L*. *iners*. Due to limited numbers of specimens dominated by *L*. *gasseri* (n = 6) or *L*. *jensenii* (n = 10), we combined these groups for statistical analysis. As only one specimen was dominated by *B*. *breve* this was combined with the heterogeneous non-lactobacilli for analysis. The majority of BV samples clustered to the community state groups dominated by *G*. *vaginalis*.

#### Factors associated with dominant bacterial community type and consistency over time

S1 Table shows the distribution of specimens within each of the subsequent five community groups by sexual activity category. By multivariate multinomial regression, Caucasian women were significantly more likely to have a vaginal microbiota dominated by *L*. *crispatus* compared to other heterogeneous non-lactobacilli, after adjusting for OCP-use and sexual activity category (ARRR = 7.28, 95%CI: 1.37, 38.57, *p* = 0.020; Table 2). Women who engaged in penile-vaginal sex were more likely to have specimens dominated by *L*. *iners* or *G*. *vaginalis* compared to other heterogeneous non-lactobacilli after adjusting for ethnicity and OCP-use (ARRR = 17.51, 95%CI: 2.18, 140.33, *p =* 0.007 and ARRR = 14.03, 95%CI: 1.22, 160.69, *p* = 0.034 respectively; Table 2).

**Table 2:** Multinomial logistic regression of factors associated with having a vaginal microbiome dominated by different bacterial species.

Outcome by dominant bacteria		Relative risk ratio (RRR)	95% CI	*p* value^a^	Adjusted RRR	95% CI	*p* value^b^
***L*. *crispatus vs* other non-lactobacilli**							
	Caucasian^c^	9.71	1.90, 49.65	**0.006**	7.28	1.37, 38.57	**0.020**
	Continuous OCP-use^d^	0.67	0.11, 4.13	0.663	0.14	0.01, 1.31	0.084
*Sexual activity category*							
	Non-coital activities only^e^	0.90	0.14, 5.85	0.916	0.51	0.84, 3.04	0.456
	Penile-vaginal sex^e^	7.86	1.01, 8.21	**0.048**	11.21	0.93, 134.74	0.057
***L*. *iners vs* other non-lactobacilli**							
	Caucasian^c^	2.34	0.49, 11.30	0.288	1.71	0.34, 8.53	0.512
	Continuous OCP-use^d^	0.60	0.10, 3.52	0.567	0.16	0.02, 1.10	0.063
*Sexual activity category*							
	Non-coital activities only^e^	1.75	0.31, 10.12	0.532	0.94	0.19, 4.76	0.942
	Penile-vaginal sex^e^	11.00	1.71, 70.73	**0.012**	17.51	2.18, 140.33	**0.007**
***L*. *gasseri* or *L*. *jensenii***^**f**^ ***vs* other non-lactobacilli**							
	Caucasian^c^	2.43	0.36, 16.22	0.360	1.88	0.24, 14.76	0.548
	Continuous OCP-use^d^	0.76	0.10, 5.97	0.792	0.24	0.02, 2.50	0.233
*Sexual activity category*							
	Non-coital activities only^e^	0.20	0.02, 1.78	0.149	0.12	0.01, 1.13	0.063
	Penile-vaginal sex^e^	2.20	0.23, 20.70	0.491	3.00	0.30, 30.16	0.350
***G*. *vaginalis vs* other non-lactobacilli**							
	Caucasian^c^	1.32	0.20, 8.71	0.770	0.93	0.12, 7.49	0.949
	Continuous OCP-use^d^	0.51	0.05, 5.22	0.573	0.11	0.01, 1.22	0.072
*Sexual activity category*							
	Non-coital activities only^e^	0.50	0.08, 3.29	0.471	0.25	0.04, 1.52	0.132
	Penile-vaginal sex^e^	7.33	0.84, 63.74	0.071	14.03	1.22, 160.69	**0.034**

^a^Multinomial logistic regression with specimens dominated by other heterogeneous non-lactobacilli (predominately *A*. *vaginae* and *Prevotella* spp.) as the referent group, clustered for multiple specimens from participants (52 clusters). Outcome refers to a composite outcome whereby the relative risk of having a specimen dominated by each group were compared simultaneously using multinomial logistic regression models

^b^multinomial logistic regression as before, adjusting for all other characteristics analysed

^c^relative to non-Caucasian women which included women from Central or South-East Asia, India or Sri-Lanka

^d^specimens were from women who reported continuous oral contraceptive pill (OCP) use at each interval of follow-up to study endpoint, relative to non-OCP users

^e^unprotected penile-vaginal sex relative to no sexual activity

^f^specimens dominated by *L*. *gasseri* and *L*. *jensenii* were combined for analysis due to smaller sample sizes for both spp.

**Fig 3:**
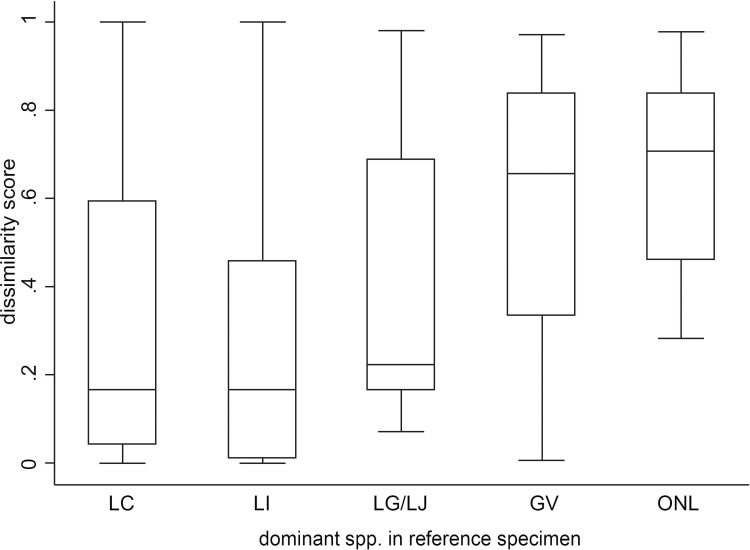
Distribution of Bray-Curtis dissimilarity scores for specimens dominated by one of five species groups. Bray-Curtis dissimilarity scores were calculated between consecutive paired longitudinal samples over multiple time points from each participant (i.e. enrolment specimen [reference] vs month 3, month 3 [reference] vs month 6 etc.). Boxplots demonstrate the distribution of Bray-Curtis dissimilarity values (box = interquartile range; black line in box = median value; T bars = range of values) for each reference specimen dominated by *Lactobacillus crispatus* (LC), *L*. *iners* (LI), other *Lactobacillus* spp. (*L*. *gasseri* or *L*. *jensenii;* LG/LJ), *Gardnerella vaginalis* (GV), or other heterogeneous non-lactobacillus spp. (primarily *Atopobium vaginae* and *Prevotella* spp.; ONL).

The median dissimilarity scores for pairs of consecutive specimens in which the first specimen (reference specimen) was dominated by *L*. *crispatus*, *L*. *iners*, other lactobacilli, *G*. *vaginalis* or other non-lactobacilli were 0.17, 0.17, 0.22, 0.66, 0.70, respectively (Fig 3). The lower scores indicate that in pairs of consecutive specimens, if the reference specimen was dominated by *L*. *crispatus*, *L*. *iners* or other lactobacilli the subsequent specimen was more likely to contain the same species in contrast to reference specimens that were dominated by other non-lactobacilli (*p*<0.001, *p*<0.001, *p* = 0.030 respectively; Table 3). This reflects consistency over time for those dominated by lactobacilli. Self-reported ethnicity, OCP-use and sexual activity did not significantly influence the consistency of the vaginal bacterial spp. present over the 3-month time period between paired specimens from an individual (Table 3).

**Table 3:** Bacterial consistency determined by Bray-Curtis dissimilarity scores between consecutive pairs of specimens.

Characteristic		Mean difference in dissimilarity scores within each characteristic (adjusted for all covariates)	95% CI	*p* value^a^
	Caucasian ethnicity^b^	-0.04	-0.17, 0.08	0.492
	Continuous OCP-use^c^	-0.12	-0.24, 0.01	0.063
Sexual activity category^d^				
	No sex during paired intervals	ref		
	Non-coital/initiate non-coital	-0.05	-0.19, 0.09	0.492
	Penile-vaginal sex/initiate penile-vaginal sex	0.06	-0.06, 0.19	0.307
Dominant bacteria in reference specimen^e^				
	Other heterogeneous non-lactobacilli	ref		
	*L*. *crispatus*	-0.36	-0.53, -0.19	**<0.001**
	*L*. *iners*	-0.38	-0.53, -0.23	**<0.001**
	*L*. *gasseri* or *L*. *jensenii*	-0.25	-0.48, -0.02	**0.030**
	*G*. *vaginalis*	-0.15	-0.38, -0.02	0.197

Bray-Curtis dissimilarity scores were calculated between paired consecutive longitudinal samples over multiple time points from each participant, where the first sample in each consecutive pair is the reference specimen. Scores were given a value from 0–1; samples with a score of 0 being having all species/genera in common and those scoring 1 with no bacterial species/genera in common.

^a^regression analysis using a random-effects model adjusting for all listed characteristics and clustering by participant to account for multiple specimens (52 clusters)

^b^compared to the combined ethnicity groups Central or South-East Asian and Indian or Sri-Lankan

^c^compared to non-oral contraceptive pill (OCP) users

^d^sexual activity categories for this analysis were specified as non-coital experience or progression from no activity to non-coital sex or unprotected penile-vaginal sex or initiation of unprotected penile-vaginal sex throughout the follow up period and both were compared to women who had no sex during the paired intervals

^e^compared to first specimen of each consecutive pair (reference specimen) dominated by other heterogeneous non-lactobacilli (predominately *A*. *vaginae* and *Prevotella* spp.)

#### *G*. *vaginalis* detection and clade distribution

*G*. *vaginalis* was detected in 77 (42%) unique specimens and in at least one longitudinal specimen provided by 35 (67%) of the participants. Sixty-five *G*. *vaginalis* positive specimens (84%) from 32 women were assigned a clade (Table 4). Of the 65 specimens 15 were from ten women with no history of sexual activity (neither non-coital nor penile-vaginal). In addition, 16 specimens were from eleven women who engaged in only non-coital activities during the preceding 3-month interval and 34 specimens were from eleven women who engaged in penile-vaginal sex in the preceding 3-month interval. No specimens contained all four clades. There was no difference in the clade type between specimens from women reporting no history of penile-vaginal sex or non-coital sex only, so these were combined for analysis. The number of specimens containing GV1/2 or 3 did not significantly differ between women with no history of penile-vaginal sex compared to women who engaged in penile-vaginal sex or specimens from women who developed BV. Specimens (pre-BV or BV) from women who went on to develop BV were more likely to contain GV4 (OR = 11.82, 95%CI: 1.87, 74.82, *p* = 0.009; Table 4). Furthermore, specimens were more likely to contain multiple *G*. *vaginalis* clades rather than a single clade if women engaged in penile-vaginal sex (3 vs 1 clade: RRR = 9.55, 95%CI: 1.33, 68.38, *p* = 0.025) or were diagnosed with BV (2 vs 1 clade: RRR = 11.82, 95%CI: 1.87, 74.82, *p* = 0.009; 3 vs 1 clade: RRR = 31.50, 95%CI: 1.69, 586.87, *p* = 0.021; Table 4).

**Table 4:** *Gardnerella vaginalis* clade distribution according to sexual activity category or BV status (N = 65 specimens, 32 women).

***G*. *vaginalis* clades detected**^a^	Sex activity or BV group^b^	n, N (%)	OR (95% CIs)	*p* value^c^
GV1	No sex/NC	19, 31 (61.3)	1.00	
	PVS	12, 19 (63.2)	1.08 (0.26, 4.52)	0.913
	BV	14, 15 (93.3)	8.84 (0.77, 101.42)	0.080
GV2	No sex/NC	12, 31 (38.7)	1.00	
	PVS	7, 19 (36.8)	0.92 (0.22, 3.81)	0.913
	BV	4, 15 (26.7)	0.56 (0.15, 2.28)	0.432
GV3	No sex/NC	ND		
	PVS	1, 19 (5.3)	*omitted*	
	BV	ND		
GV4	No sex/NC	11, 31 (35.5)	1.00	
	PVS	12, 19 (63.2)	3.12 (0.76, 12.80)	0.115
	BV	13, 15 (86.7)	11.82 (1.87, 74.82)	**0.009**
**No. of *G*. *vaginalis* clades detected**	Sex activity or BV group^b^	n (%)	RRR (95% CIs)	*p* value^d^
Single clade	No sex/NC	21, 31 (67.7)		
	PVS	11, 19 (57.9)	*ref*	
	BV	2, 15 (13.3)		
2 clades	No sex/NC	9, 31 (29.1)	1.00	
	PVS	3, 19 (15.8)	0.64 (0.11, 3.58)	0.608
	BV	10, 15 (66.7)	11.67 (1.44, 94.20)	**0.021**
3 clades	No sex/NC	1, 31 (3.2)	1.00	
	PVS	5, 19 (26.3)	9.55 (1.33, 68.38)	**0.025**
	BV	3, 15 (20.0)	31.50 (1.69, 586.98)	**0.021**

^a^Specimens that were positive for *Gardnerella vaginalis* were analysed by clade-specific qPCR to characterise the *G*. *vaginalis* clade present (GV1/2/3/4)

^b^There was no difference in clade type between specimens from women reporting no sexual activity or non-coital sex only (No sex/NC) so these were combined for analysis and compared to specimens from women reporting unprotected penile-vaginal sex who don’t get BV (PVS) or all specimens from women who develop BV (BV)

^c^*G*. *vaginalis* clade abundances were analysed using logistic regression with women reporting no sex/non-coital sexual activity only as the referent group, clustering by participant to take into account multiple specimens provided longitudinally

^d^Multinomial logistic regression with specimens containing a single clade as the referent group, clustered for multiple specimens from participants (32 clusters). Outcome refers to a composite outcome whereby the relative risk of number of clades within each sexual activity/BV group compared simultaneously using multinomial logistic regression models. Key: ND = Not Detected, OR = odds ratio, RRR = relative risk ratio.

## Discussion

This longitudinal study examining the vaginal microbiota of young women showed that sexual activity and ethnicity influenced the dominant bacteria detected. Women engaging in unprotected penile-vaginal sex were more likely to have vaginal bacterial communities dominated by *L*. *iners* or *G*. *vaginalis* rather than other heterogeneous non-lactobacilli, after adjusting for OCP-use and sexual activity, and Caucasians were more likely to have a microbiota dominated by *L*. *crispatus* than other non-lactobacilli. Bacterial communities remained remarkably consistent over time, particularly when dominated by lactobacilli, and the dominant spp. detected over time was not influenced by sexual activity in this cohort. However, women who reported penile-vaginal sex or who developed BV were more likely to have multiple clades of *G*. *vaginalis* compared to a single clade. The presence of GV4 in a specimen was associated with developing/having BV but the presence of other clades was not associated with specific types of sexual activity.

Cluster analysis of the bacterial communities in this study revealed similar bacterial CSTs to those described in healthy reproductive-age North-American women and women of European ancestry [4, 6, 16], with the two most abundant bacterial groups being dominated by *L*. *crispatus* and *L*. *iners* [4, 6, 16, 17]; both of which we found to consistently dominate specimens from the same individual over time in our study. *L*. *crispatus* is thought to play a key role in host defence due to a high production of lactic acid [18], a known inhibitor of BV [19]. It dominates the vaginal microbiota in reproductive years when elevated oestrogen levels, together with lactic acid production, support a glycogen-rich vaginal epithelium [20]. In contrast, *L*. *iners* dominated women have lower concentrations of D-lactic acid and women diagnosed with BV by standard clinical or laboratory criteria can have specimens that are dominated by this spp. [3, 18]. A study of *L*. *iners* strains found that several proteins detected in other vaginal lactobacilli were absent in the *L*. *iners* genomes. This could explain why *L*. *iners* may contribute to BV development [21]. Although few participants reported penile-vaginal sexual debut, *L*. *iners* dominated in those engaging in penile-vaginal sex, suggesting initiation of penile-vaginal sex may affect the vaginal microbiota. This is in contrast to the study by Mitchell *et al*. [1], in which women who initiated sexual activity had no change in colonisation by any *Lactobacillus* spp.

The other determinant of dominant bacterial spp. in this study was ethnicity. Caucasian women and not women of Central and South-East Asian or Indian/Sri-Lankan ethnicities, were more likely to harbour a *L*. *crispatus*-dominated vaginal microbiota than one dominated by heterogeneous non-lactobacilli, independent of sexual activity or OCP-use. Previous studies have consistently demonstrated an association between ethnicity and vaginal microbiota [4, 6], in particular the association between Caucasian ethnicities and *Lactobacillus* spp. [6].

As the majority of published studies report cross-sectional data, analyses of longitudinal changes in the vaginal microbiota of young women are limited. Longitudinal studies that have sampled older and predominantly sexually-active women have shown that while the vaginal microbiota can be quite stable its composition can also fluctuate quite rapidly and be influenced by a range of factors including menses, sexual behaviour and antimicrobials [5, 15, 22–24]. We found if a woman’s reference specimen was dominated by a lactobacilli spp. then her subsequent samples was more likely to contain the same spp., indicating these species were associated with consistency or stability of the vaginal microbiota over 3 month intervals. Unfortunately, we were unable to establish if the women in our cohort dominated by lactobacilli spp. were more likely to have partner consistency between intervals as we were limited by the small numbers of participants in specific groupings. However, our findings are supported by previous studies which suggest that women with an *L*. *crispatus* dominated microbiota are more likely to remain stable over time [5, 15, 22]. Our findings contrast however with other data indicating a *L*. *iners* dominated vaginal microbiota is more likely to be associated with a vaginal microbiota that fluctuates over time [5]. One reason for this discrepancy is the 3 month interval between samples may have resulted in us missing more transient or rapid fluctuations, but could also be due to the lower risk profile of our younger population sampled.

In this study OCP-use did not appear to influence bacterial community type or the consistency of dominant spp. detected between sequential intervals. Hormonal contraceptive use has however been associated with a reduced risk of BV in a meta-analysis of observational studies [25]. One hypothesized mechanism is that oestrogen-containing OCPs increase the glycogen content of vaginal epithelial cells, supporting growth of favourable lactobacilli including *L*. *crispatus*, increasing lactic acid concentrations and inhibiting the growth of BV-associated bacteria [19]. Our findings may be explained by the small numbers included in this analysis or the low rate of OCP use (24%) in our study population. Furthermore, the potential confounding effect of OCP-use in this study may have been minimized as not all OCPs contain oestrogen and the formulations used were not recorded.

Similarly to Mitchell et al (2012) who reported that initiation of penile-vaginal sex resulted in increased colonization with *G*. *vaginalis* [1], we showed that engaging in penile-vaginal sex was associated with having a vaginal microbiota dominated by *G*. *vaginalis*. We also found that engaging in unprotected penile-vaginal sex was associated with the acquisition of multiple clade types. This suggests that commensal clades of *G*. *vaginalis* [9], including potentially pathogenic and antibiotic-resistant clades [26], could be transmitted to women through penile-vaginal sex. Specimens from women with BV were more likely to have GV4 present and multiple clades present than women who had not engaged in penile-vaginal sex. This is supported by studies showing male carriage of *G*. *vaginalis* [27, 28], and that the bacterial composition of the penile coronal sulcus microbiota is influenced by both circumcision and sexual activity [29, 30]. Examination of specific *G*. *vaginalis* clades in men would assist in our understanding of their role as commensal organisms and in the pathogenesis of BV.

There were several limitations to this study. In the overall FUSS cohort, participants were highly educated with low numbers initiating sexual activity, smoking or douching and BV was rare (n = 7/413 cases). These women were younger on average than other populations and thus had fewer numbers of lifetime sexual partners if any at all. Therefore our findings may not be generalizable to other populations of young women. Participants self-collected vaginal swabs and were not examined by a physician and diagnosis of BV was restricted to the Nugent method. Because specimens were collected every three months and menstrual data not recorded, we may have missed interval cases of BV and/or short-term fluctuations in bacterial diversity that could have been influenced by menses [5]. The presence of small numbers of women in some of the subgroups, and the low BV incidence rate may have limited analyses. Ideally we would have analysed differences in the microbiota between women practicing protected and unprotected sex however, we were limited by the small number of participants that reported 100% condom use for all penile-vaginal sex in an interval. This prevented us conducting an analysis exclusively based on condom use for penile-vaginal sex. The association between ethnicity and dominant spp. may have been confounded by contraceptive and sexual practices, but we included these factors in all adjusted analyses. Finally, as with studies incorporating 16S rRNA gene-based pyrosequencing, there is the potential for PCR-based methodologies to fail to amplify certain bacterial members, and taxonomic resolution limited in species with highly similar 16S rRNA gene sequences [31]. Thus we validated our 16S rRNA gene amplicon dataset based on the V3/4 variable regions with a subset analysis of V1/2 sequencing and qPCR screening for *L*. *crispatus*, *L*. *iners* and *G*. *vaginalis*.

### Conclusions

In this longitudinal study of young women with a range of sexual experience, ethnicity and engaging in penile-vaginal sex influenced the composition of the vaginal microbiota. Overall, there was a very high level of consistency of vaginal bacterial communities between consecutive pairs of specimens over 12 months, particularly when either *L*. *crispatus*, *L*. *iners* or *L*. *gasseri* or *L*. *jensenii* were the dominant bacterial species present in the reference specimen. Although engagement in penile-vaginal sex did not appear to result in a persistent change in the consistency of vaginal microbial communities over time, it was associated with increased *G*. *vaginalis* clade diversity in young women with and without BV, supporting sexual transmission of commensal and potentially pathogenic clades of *G*. *vaginalis*.

## Supporting information

S1 Table Number of specimens from women by each dominant bacterial spp. and each sexual activity category. (PDF)
