# Supplementary material for: The influence of sexual activity on the vaginal microbiota and Gardnerella vaginalis clade diversity in young women
Source: PLoS One. 2017 Feb 24;12(2):e0171856. doi: 10.1371/journal.pone.0171856 (PMC5325229; doi:10.1371/journal.pone.0171856)
Supplement: S1 Table — (PDF) [file pone.0171856.s001.pdf]

Supplementary Table 1. Number of specimens from women by each dominant bacterial spp. and each sexual activity category

**Dominant bacterial spp. in each specimen**[a]	**Sexual activity category, n (%)**			
	no sexual activity (N=60)	non-coital activities (N=53)	penile-vaginal sex (N=68)	Total specimens per dominant spp. group, n (%) (N=184)
L. crispatus	21 (30)	19 (27)	30 (43)	70 (38)
L. iners	12 (21)	21 (37)	24 (42)	57 (31)
L. gasseri or *L. jensenii*[b]	10 (63)	2 (13)	4 (25)	16 (9)
G. vaginalis	6 (35)	3 (18)	8 (47)	17 (9)
Other heterogeneous non-lactobacilli spp.[c]	11 (46)	11 (46)	2 (8)	24 (13)

[a]The median abundance of each dominant spp. was as follows: *L. crispatus* 96.2% (IQR=83.2-98.7), *L. iners* 91.4% (IQR=76.3-99.3), other *Lactobacillus* spp. 75.8% (IQR=53.2-88.4), *G. vaginalis* 65.6% (IQR=57.3-85.0), other heterogeneous non-lactobacilli spp. 51.4% (IQR=36.9-74.6); [b]*L. gasseri* and *L. jensenii* dominant specimens were combined due to lower numbers in both groups; [c]other heterogeneous non-lactobacilli spp. were predominately *Atopobium vaginae* and *Prevotella* spp.
